# The Influence of Task-Irrelevant Flankers Depends on the Composition of Emotion Categories

**DOI:** 10.3389/fpsyg.2016.00712

**Published:** 2016-05-25

**Authors:** Barbara Schulte Holthausen, Christina Regenbogen, Bruce I. Turetsky, Frank Schneider, Ute Habel

**Affiliations:** ^1^Department of Psychiatry, Psychotherapy and Psychosomatics, Medical Faculty, RWTH Aachen UniversityAachen, Germany; ^2^JARA – BRAIN Institute 1: Structure Function Relationship, RWTH Aachen UniversityAachen, Germany; ^3^Department of Clinical Neuroscience, Karolinska InstitutetStockholm, Sweden; ^4^Department of Psychiatry, University of Pennsylvania, PhiladelphiaPA, USA

**Keywords:** threat superiority, distractor, congruency, gender differences, emotional face processing, flanker, stimulus onset asynchrony

## Abstract

Face recognition usually takes place in a social context, where faces are surrounded by other stimuli. These can act as distracting flankers which impair recognition. Previous work has suggested that flankers expressing negative emotions distract more than positive ones. However, the various negative emotions differ in their relative impact and it is unclear whether all negative emotions are equally distracting. We investigated the impact of three negative (angry, fearful, sad) and one positive (happy) facial flanker conditions on target recognition in an emotion discrimination task. We examined the effect of the receiver’s gender, and the impact of two different temporal delays between flanker and target onset, as stimulus onset asynchrony is assumed to affect distractor strength. Participants identified and rated the emotional intensity of target faces surrounded by either face (emotional and neutral) or non-face flankers. Target faces were presented either simultaneously with the flankers, or delayed by 300 ms. Contrary to our hypothesis, negative flankers did not exert stronger distraction effects than positive or neutral flankers. However, happy flankers reduced recognition performance. Results of a follow-up experiment with a balanced number of emotion categories (one positive, one negative and one neutral flanker condition) suggest that the distraction effect of emotional flankers depends on the composition of the emotion categories. Additionally, congruency effects were found to be valence-specific and overruled by threat stimuli. Females responded more quickly and rated targets in happy flankers as less intense. This indicates a gender difference in emotion processing, with greater sensitivity to facial flankers in women. Targets were rated as more intense when they were presented without a temporal delay, possibly due to a stronger flanker contrast. These three experiments show that an exceptional processing of threat-related flanker stimuli depends on emotion category composition, which should be considered a mediating factor when examining emotional context effects.

## Introduction

Our ability to rapidly infer someone else’s mental state by deciphering their facial expression is an essential prerequisite for successful human social interactions, which serve adaptive purposes (Ekman and Friesen, 1971; Shariff and Tracy, 2011). Studies investigating facial emotion processing have typically employed standardized batteries of isolated static faces (Adolphs, 2002). But because isolated faces do not capture the complexity present in our everyday social interactions, researchers have begun to study contextual influences on face and emotion perception (Righart and de Gelder, 2008b; Van den Stock and de Gelder, 2012). Several studies have confirmed a biasing effect by contextual information originating from multiple sources, e.g., background scenes (Righart and de Gelder, 2006; den Stock et al., 2014), body expressions (Van den Stock et al., 2007), or faces (Lavie et al., 2003). Distraction effects caused by context are reported to be automatic and unintentional (Aviezer et al., 2011; Van den Stock and de Gelder, 2014), and stronger when contextual stimuli are of social relevance (Lavie et al., 2003).

Contextual stimuli communicating threat (e.g., anger or fear) seem to be advantageous in capturing attention, which might be explained by evolutionary mechanisms regarding the importance of threat detection. The latter is supported by results on the Face-in-the-Crowd (FITC) visual search task. This task demands participants to detect the unique emotional expression (e.g., anger) in a crowd of emotionally homogenous distractor faces. In line with this, an angry target face is identified faster and more accurate when surrounded by neutral or emotional distractor faces compared to happy or neutral targets (Hansen and Hansen, 1988; Eastwood et al., 2001; Öhman et al., 2001). An alternate explanation for a threat-specific superiority suggests low-level perceptual differences responsible for “pop-out” effects. This assumes that angry faces draw attention due to their discontinuity with the lower face boundaries (chin, upward u-shape) compared to happy face shapes, which are more congruent with the general face boundaries (Coelho et al., 2010; Purcell and Stewart, 2010). The interpretation is mainly supported by studies using simple schematically drawn faces. Yet, the emotional impact might be insufficient to induce any measureable attentional shift, which makes it difficult to draw conclusions (Schmidt and Schmidt, 2013).

Generally, most studies investigating whether or not threatening faces draw more attention than other emotion categories have used the FITC task. In contrast, the flanker task measures response interference when a centrally presented target stimulus is surrounded by different irrelevant flankers (Eriksen and Eriksen, 1974). Originally, the stimuli consisted of letters, but were extended to emotional faces (Fenske and Eastwood, 2003). Here, negative target faces captured subjects’ attention more than positive or neutral ones; hence flanker stimuli did not exert a comparable distraction in negative targets. This was explained by a narrowing of attention in stimuli with a negative valence. Results were, however, limited to one single negative emotion, i.e., sadness, which was compared to the attention capture of happy and neutral faces. It remains unclear whether the observed effect is generalizable to facial flankers – e.g., whether negative flanker faces capture more attention than happy or neutral ones (‘distractor hypothesis’). Furthermore, it is of interest whether all negative emotions distract to the same extent or whether this is most evident for stimuli signaling fear or anger (Stenberg et al., 1998).

To investigate whether specific emotions within the negative emotion range result in stronger interference effects, we used facial flankers of either one of three negative or one positive emotion, and assessed their effects on concurrently presented target faces. We hypothesized that emotion recognition performance would be slower and less precise for targets presented with threat-expressing flankers (anger and fear) than with flankers expressing other negative (sadness), positive or neutral emotions. This may be explained by the distractor hypothesis, stating that evolutionary salient cues capture attention and distract from the target. It is, however, also conceivable that congruency effects influence the expected results: there is evidence that congruent target-flanker combinations predict better performance than incongruent ones (‘congruency hypothesis,’ Gelder et al., 2006; Kret and Gelder, 2010; Ito et al., 2012). Yet the picture is even more complex and suggests an interaction between valence and congruence: a few studies reported valence differences with stronger congruency effects in positive compared to negative target-flanker combinations (Fenske and Eastwood, 2003; Righart and de Gelder, 2008a,b).

The influence of emotional flanker faces may be different between males and females. On the one hand, women generally perform better in emotion recognition tasks and rate emotions as more intense (Fujita et al., 1991; Hall and Matsumoto, 2004; but see Barrett et al., 1998). On the other hand, women seem to pay more attention to peripheral stimuli, evident by stronger cuing effects to neutral objects as well as greater distractibility by irrelevant facial features (Merritt et al., 2007; Stoet, 2010; Feng et al., 2011). More specifically, women are reported to be more influenced by positive emotional primes only (Donges et al., 2012). Based on these inconsistent results, we considered whether potential differences between men and women in recognition accuracy and reaction times are valence-specific.

Besides the emotional impact of the flanker, the temporal dynamics of stimulus presentation [i.e., the stimulus onset asynchrony (SOA) between flanker and target stimulus presentation] are known to influence target face processing. Previous work suggests that the interference effect is strongest when target and flanker are presented simultaneously and decreases rapidly with an increasing delay between flanker and target. With SOA greater than 200 ms, the distracting effect of preceding flankers is thought to be relatively small (Taylor, 1977; Botella et al., 2002). We therefore tested two different flanker-target timing modes. In Experiment 1, we presented flankers and targets simultaneously (SOA 0 ms). In Experiment 2, the onset of the flankers preceded the central targets by 300 ms (SOA 300 ms). We expected antecedent flankers to result in higher accuracy and intensity ratings of target faces due a weaker interference effect, compared to a simultaneous onset of flanker and target (SOA 0 ms).

The aim of the present study was to investigate whether an influence of task-irrelevant facial flankers can be explained by the ‘distractor hypothesis’ (worse performance when targets are surrounded by threat-associated flankers). Our approach also enabled to test whether results fit the ‘congruency hypothesis’ and to take into account potential valence-specificity for congruency. Gender differences as well as temporal dynamics (SOA) were additionally taken into consideration, due to their previously reported interferring influence.

## Materials and Methods

Two separate experiments were conducted, one with flanker and target faces presented simultaneously (Experiment 1, SOA 0 ms), the other one with the onset of flankers presented prior to the onset of the target (Experiment 2, SOA 300 ms). All other aspects (material and stimuli, task) were held constant across the two experiments. The studies were performed in accordance to the Declaration of Helsinki and approved by the local institutional review board (protocol number EK 040/12). Participants were informed about the study protocol and gave written informed consent. They were reimbursed with 10 Euro.

### Material and Stimuli

Pictures of 10 males and 10 females characters were selected from a standardized stimulus set (Gur et al., 2002). They were comparable in age, emotion intensity, emotion valence, and visual luminance.

**FIGURE 1:**
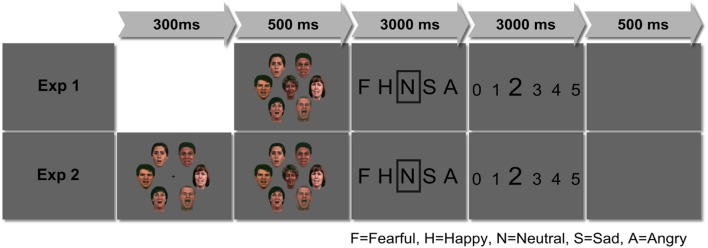
**Experimental design set-up.** In Experiment 1, flanker-target combinations were presented simultaneously for 500 ms, in Experiment 2, flanker presentations preceded the target face by 300 ms, then, flankers and targets were also presented simultaneously for 500 ms.

Stimuli in both experiments consisted of color pictures of a centrally presented face (‘target’) surrounded by six other faces (‘flanker’), all presented on gray background (**Figure 1**). Target and flanker faces each consisted of five different emotion categories (fearful, sad, angry, happy, and neutral). Additional ‘scrambled’ non-face flankers were included as a control condition (created with mosaic filter in Adobe^®^ Photoshop^®^ 6.0; for example stimuli see **Supplementary Figure S1**). The gender ratio of target faces and context faces was balanced.

All faces were adjusted to a size of 3.6 cm × 5.8 cm (3.9° × 6.26°). The target face was presented in the center of the screen, the flanker faces were arranged circle-wise with a diameter of 11.73 cm (12.7°). The distance from the midpoint of the target face to the midpoint of the flanker faces was 6.4° of visual angle at a viewing distance of 53 cm (compare Fox et al., 2000; Adamo et al., 2010a,b). Stimuli were presented using Presentation^®^ (version 14, Neurobehavioral Systems, Inc., San Francisco, CA, USA).

Each experiment consisted of 520 trials. 100 different target faces (20 actors × 5 target emotions) were each shown with five different emotional flanker categories (e.g., a happy target face was surrounded by either happy, sad, neutral, angry, or fearful facial flankers). Additionally, 20 target faces were surrounded by ‘scrambled’ non-face flankers. Conditions were presented in a pseudo-randomized order. Experiments consisted of a 6 (5 flanker emotions plus scrambled condition) times 5 (target emotion) design.

### Task

In Experiment 1, target-flanker combinations were presented simultaneously for 500 ms (SOA 0 ms), followed by an emotion identification period of up to 3000 ms (**Figure 1**). In Experiment 2, the target-flanker presentation (500 ms) was preceded by the presentation of the flanker alone for 300 ms (SOA 300 ms). During this interval, a fixation cross was presented in the center of the facial flankers to announce the position of the subsequent target face and facilitate gaze fixation. After 300 ms, the fixation cross was replaced by the target face without any change in the surrounding flanker.

Apart from the difference in flanker-target presentation latency (SOA 0 ms vs. SOA 300 ms) the task was the same in both experiments: participants were instructed to identify the target face’s emotion (choosing from all presented emotions plus neutral) via keyboard button press. This was followed by a period of up to 3000 ms during which participants rated the intensity of the target emotion on a Likert scale ranging from 0 to 5. A blank screen was presented for 500 ms before the next trial onset. If participants chose ‘neutral,’ no intensity rating was prompted. Halfway through the experiment, participants could take a break for as long as they wanted.

### Participants

Experiment 1 included 28 healthy adults (14 males, *M* age = 28.2 years, *SD* = 7.9 years). Originally, 29 participants took part in Experiment 1, but one participant was excluded due to incorrect button use. Experiment 2 included a separate sample of 28 participants (14 males, *M* age = 27.8 years, *SD* = 6.7 years). The two samples did not differ significantly in age (*t*_(54)_ = 0.18, *p* = 0.86). Participants were recruited through local advertisements and were prescreened to confirm a negative life-time history of psychiatric disorder, neurological illness or current substance abuse (SKID light, Wittchen et al., 1997). All participants reported normal or corrected-to-normal vision.

### Data Analysis

Trials with omitted responses (*n* = 164) were excluded from further analysis (0.5%). All participants performed under the cut-off criterion for exclusion (10%, 52 responses; *M*_omitted_ = 3.21, *SD* = 0.06). For each flanker emotion, relative frequencies of correctly recognized trials were computed. Three repeated measures generalized estimating equations (GEEs) were carried out in IBM^®^ SPSS^®^ (version 20). GEEs account for binomial distributions, correct for potential violations of normal distribution and for non-sphericity, and they enable modeling subject effects and conditions with an unequal number of trials. Each model contained data from both experiments. All models included the within-subject factor ‘flanker emotion’ (six levels), the between-subject factor ‘SOA’ (two levels), and the between-subject factor ‘gender’ (two levels). All main effects and interactions were tested for significance.

Three models analyzed emotion recognition accuracy, intensity ratings of correct trials (excluding trials with neutral targets since there existed no intensity ratings), and reaction times for correct trials, including the above-mentioned factors.

Three additional GEEs (analyzing recognition accuracy, intensity ratings, and reaction time) were calculated to test whether ‘congruency of flanker and target’ (within-subject factor, two levels) had an influence on the participants’ performance. The factor ‘SOA’ was also included in these models, to check for potential interactions (between-subjects factor, two levels).

All *post-hoc* pairwise comparisons were corrected for multiple comparisons using Bonferroni-correction.

## Results

The overall emotion identification accuracy for the target faces was *M* = 77.43% (*SEM* = 0.13) in Experiment 1 and *M* = 76.55% (*SEM* = 0.12) in Experiment 2. The difference between the two experiments was not significant (*t*_(54)_ = 0.51, *p* = 0.61). For confidence interval sizes of all results, please see **Supplementary Tables S1**–**S6**.

### Flanker Emotion

**FIGURE 2:**
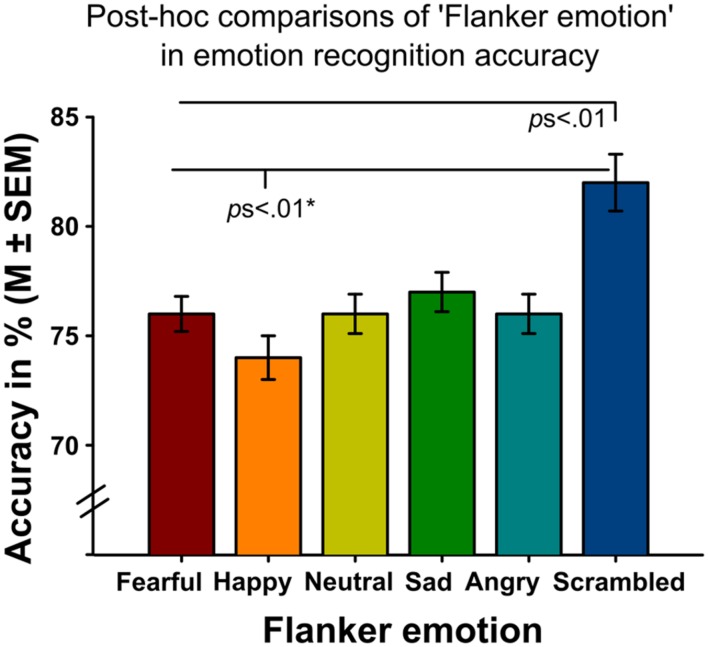
**Emotion recognition accuracy of target faces surrounded by different flanker emotions (*x*-axis) across both experiments.** The highest emotion recognition accuracy was found for targets surrounded by scrambled flankers while targets surrounded by happy flankers showed the lowest emotion recognition accuracy. Note that the comparison of happy and angry flankers only reached statistical trend level.

**FIGURE 3:**
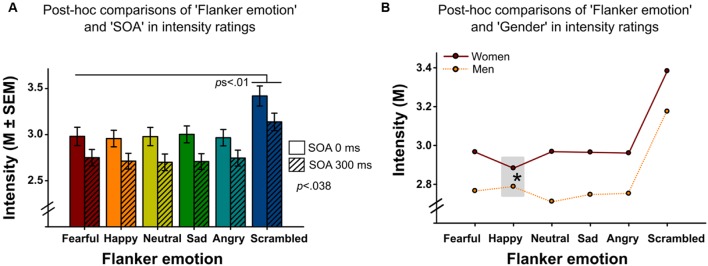
**(A)** Emotion intensity ratings of target faces surrounded by different flanker emotions (*x*-axis) in Experiments 1 and 2. Delayed flanker-target presentations in Experiment 2 (SOA 300 ms) were associated with lower intensity ratings compared to simultaneous presentations in Experiment 1 (SOA 0 ms). Targets surrounded by scrambled flankers were rated more intense than targets surrounded by facial flankers. **(B)** Mean intensity ratings of target faces surrounded by different flanker emotions (*x*-axis) in women and men. Females rated targets surrounded by happy flankers significantly less intense than males (*post-hoc* comparison of the interaction between the within-subject factor ‘flanker emotion’ and the between-subject factor ‘gender’ indicated by the asterisk).

Analyses revealed significant main effects of ‘flanker emotion’ on emotion recognition accuracy (Wald-Chi^2^_(5)_ = 46.109, *p <* 0.001, **Figure 2**), intensity ratings (Wald-Chi^2^_(5)_ = 113.01, *p <* 0.001) and reaction times (Wald-Chi^2^_(5)_ = 17.943, *p* = 0.003). *Post-hoc* pairwise comparisons indicated that targets surrounded by scrambled flankers were recognized better (all *p*s < 0.01), but more slowly (only the comparison between scrambled and sad flankers survived Bonferroni-correction, *p* = 0.039). Targets surrounded by scrambled flankers were additionally rated as more intense than targets surrounded by facial flankers (all *p*s *<* 0.01, **Figure 3A**). Target recognition was less accurate when surrounded by happy flankers (all *p*s < 0.01, except for angry vs. happy, *p* = 0.062).

### Stimulus Onset Asynchrony

The main effect of ‘SOA’ reached significance for emotion intensity ratings (Wald-Chi^2^_(1)_ = 4.297, *p* = 0.038, **Figure 3A**), with higher target intensity ratings following simultaneous (SOA 0 ms, Experiment 1) compared to antecedent (SOA 300 ms, Experiment 2) presentations. Other main effects did not reach significance (SOA on emotion recognition accuracy: Wald-Chi^2^_(1)_ = 0.295, *p* = 0.59, SOA on reaction time: Wald-Chi^2^_(1)_ = 0.8, *p* = 0.37).

### Gender

**FIGURE 4:**
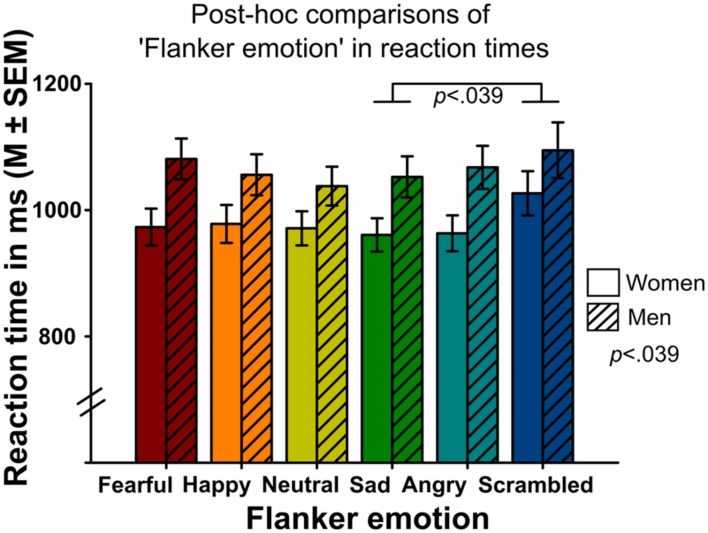
**Mean reaction times of correct responses surrounded by different flanker emotions (*x*-axis) in women and men.** Women were faster in emotion recognition than men (main effect of the between-subject factor ‘gender’). Targets in scrambled flankers were recognized slower compared to targets surrounded by sad flankers.

Analyses indicated a significant main effect of ‘gender’ on reaction time (Wald-Chi^2^_(1)_ = 4.275, *p* = 0.039, **Figure 4**), with faster reaction times in women compared to men. Other main effects did not reach significance (gender effect on emotion recognition accuracy: Wald-Chi^2^_(1)_ = 0.04, *p* = 0.84, gender effect on intensity ratings: Wald-Chi^2^_(1)_ = 2.51, *p* = 0.11).

The interaction of ‘gender’ by ‘flanker emotion’ was also significant (Wald-Chi^2^_(5)_ = 27.376, *p <* 0.001, **Figure 3B**). Women rated targets surrounded by happy flankers as least intense, while men rated them as second most intense. Other interactions did not reach significance.

### Congruency

Neither the main effect of ‘congruency’ nor any of the interactions with ‘SOA’ reached significance when testing the influence of ‘congruency’ on emotion recognition accuracy, intensity ratings, and reaction time (emotion recognition: Wald-Chi^2^_(1)_ = 1.31, *p* = 0.25, intensity ratings: Wald-Chi^2^_(1)_ = 1.06, *p* = 0.30, reaction time: Wald-Chi^2^_(1)_ = 2.87, *p* = 0.09).

All analyses were run additionally without the scrambled flanker condition and revealed similar results.

## Experiment 3

The results from Experiments 1 and 2 provoked the question whether the unbalanced composition of negative and positive emotions (three negative flanker emotions, one positive, one neutral emotion) might be responsible for the unexpected, less accurate identification of targets in happy flankers. As the unbalanced composition was one of the major differences in comparison to earlier studies, we wanted to specifically assess the effect of emotion category composition on target emotion recognition accuracy, intensity ratings and reaction time, respectively in a valence-balanced design.

### Methods

A third experiment was carried out using a balanced composition of emotion categories (one positive, one neutral, and one negative flanker condition, as well as scrambled non-face flankers). The experiment consisted of 480 trials. Happy, neutral, or fearful target faces were surrounded by either happy, neutral, fearful, or scrambled non-face flankers. In opposite to Experiments 1 and 2 participants rated the target emotion on a 7-point scale ranging from fearful through neutral to happy. Time until first button press was considered as an approximation of reaction time. First reactions <150 ms were excluded. Apart from these differences, material, design, and task were the same as in Experiment 2 (SOA 300 ms).

Experiment 3 included 29 healthy adults (15 males, *M* age = 26.1 years, *SD* = 5.37), conforming to the same inclusion criteria as in Experiments 1 and 2.

Three GEEs repeated measures with the within-subject factor ‘flanker emotion’ (four levels) were calculated (analyzing the outcome variables recognition accuracy, intensity ratings, and reaction time) as well as paired *t*-tests analyzing congruency effects (for the same outcome variables). Due to their binomial distribution hit rates of emotion recognition accuracy were 2arcsin-transformed for the paired *t*-tests.

### Results

#### Flanker Emotion

**FIGURE 5:**
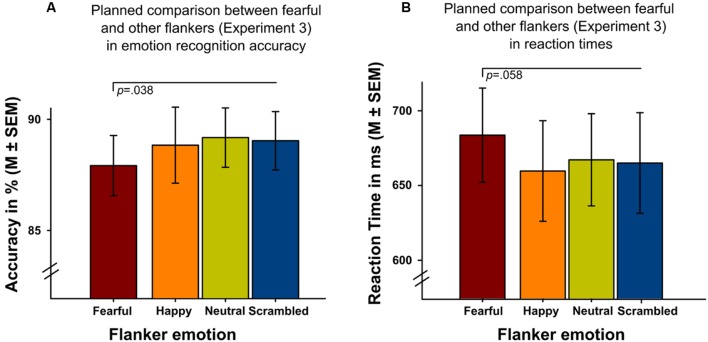
**Experiment 3 investigating emotion recognition accuracy and reaction times in target faces surrounded by four different flanker conditions (one positive, one negative, neutral and scrambled, *x*-axis).** Planned *t*-tests showed **(A)** reduced emotion recognition accuracy (*p* = 0.038) and **(B)** longer reaction times (*p* = 0.058) in targets presented with fearful facial flankers compared to targets presented with other flankers.

Analyses revealed a significant main effect of ‘flanker emotion’ on intensity ratings (Wald-Chi^2^_(3)_ = 10.995, *p* = 0.012), replicating the results of the initial experiments, with higher intensity ratings of targets in scrambled flankers. Only the *post-hoc* comparison between scrambled and neutral flankers survived Bonferroni-correction (*p* = 0.006). Flanker emotion did not show significant main effects on emotion recognition accuracy (Wald-Chi^2^_(3)_ = 6.027, *p* = 0.11) or reaction time (Wald-Chi^2^_(3)_ = 4.296*, p* = 0.231) in a balanced design. On a descriptive level (**Figure 5**) it could be seen, however, that targets surrounded by fearful flanker faces were identified less accurately (**Figure 5A**) and more slowly (**Figure 5B**), compared to all other flanker conditions. Planned paired *t*-tests between targets surrounded by fearful flanker faces compared to all other flankers (average) were significant for emotion recognition accuracy (*t*_(28)_ = 2.18, *p* = 0.038) and revealed a statistical trend for reaction time (*t*_(28)_ = 1.98, *p* = 0.058) (**Figure 5**).

#### Congruency

Results revealed that congruent target-flanker combinations were significantly better recognized than incongruent combinations (*t*_(28)_ = 2.13, *p* = 0.042). Intensity ratings and reaction times did not differ significantly between congruent and incongruent combinations (intensity ratings*: t*_(28)_ = 0.31, *p* = 0.76, reaction time: *t*_(28)_ = 1.52, *p* = 0.139).

Additionally, we performed congruency analyses separately for each emotion as previous literature occasionally reported valence-specificity of congruency effects (Fenske and Eastwood, 2003; Righart and de Gelder, 2008a,b). Paired *t*-tests compared congruent and incongruent target-flanker combinations separately for happy and fearful target faces. Results did not reveal significant differences of fearful faces surrounded by congruent or incongruent facial flankers for emotion recognition accuracy (*t*_(28)_ = 0.83, *p* = 0.413), intensity ratings (*t*_(28)_ = 0.1*, p* = 0.924) or reaction time (*t*_(28)_ = 0.57, *p* = 0.572). Happy targets, in opposite, were recognized faster (*t*_(28)_ = 2.37, *p* = 0.025) and trendwise better (*t*_(28)_ = 1.85, *p* = 0.074) in congruent compared to incongruent conditions (intensity ratings: *t*_(28)_ = 1.11, *p* = 0.662).

## Discussion

Two behavioral experiments investigating emotional context effects revealed reduced emotion recognition accuracy and intensity ratings of target faces embedded in facial compared to scrambled non-face flankers. Reaction times were increased for targets associated with scrambled flankers. However, contrary to our expectations, negative flankers did not have a greater deleterious effect on the accuracy or speed of emotion recognition for target faces. Rather, it was the target faces that were surrounded by happy flankers which were identified least accurately. A follow-up study (Experiment 3) tested a balanced emotion category composition; here, the results on happy flankers were comparable to those elicited by other flanker conditions. Results of this experiment suggested worse emotion recognition performance for targets surrounded by fearful flankers.

Contrary to our expectations, delayed presentation of the target (SOA 300 ms, Experiment 2) did not result in higher recognition accuracy compared to simultaneous presentation (SOA 0 ms, Experiment 1). However, simultaneously presented targets were perceived as more intense. Our results, additionally, replicate and extend previously observed gender differences in facial emotion processing (faster reaction times, Hampson et al., 2006). They provide support for a female advantage not only in response to isolated facial expressions, but also when emotional targets are surrounded by social flankers. Gender differences interacted with flanker emotion. While both genders were less accurate in identifying targets surrounded by happy flankers, only women showed additional effects on their intensity ratings.

On the most general level, these findings indicate that social flankers are more distracting than non-meaningful scrambled ones. Although the instruction was to ignore the flankers, participants appear to have automatically reoriented their attention toward socially relevant stimuli, which may serve a function as salient social distractors (Lavie et al., 2003). In hindsight, one shortcoming of our design was the absence of a ‘target-only’ condition with no flankers. Future studies should include such a condition to directly investigate the non-specific distracting effects of flanker stimuli, by comparing social and non-social flanker conditions with a non-flanker control condition.

Contrary to our hypotheses, negative facial flankers did not reduce emotion recognition performance or intensity ratings of target faces. Surprisingly, happy flankers caused the lowest emotion recognition accuracy of target faces. This partly replicates previous results stating that happy faces attract more attention when they are compared to several negative faces (Calvo and Nummenmaa, 2008). Here, the authors had applied a visual search task with faces and the advantage of happy faces was attributed to deviating low-level features of happy compared to negative emotional faces. Our data suggest that the effect of emotional flankers may depend on the specific composition of presented emotions. When using several negative emotions and one positive emotion, as we did, the happy emotion stands out more than any of the negative ones and attracts more attention than the plurality of negative emotions. However, when only one negative expression is tested during the experiment, negative facial expressions are reported to be more effective in capturing attention than positive or neutral expressions (Fenske and Eastwood, 2003). To further test this assumption, we conducted a third experiment in which the composition of emotion categories was balanced (happy, fearful, neutral, and scrambled flankers). Here, happy flankers were found to be equally distracting. Directly comparing targets after fearful flankers to an average of all other conditions revealed a stronger distraction effect for the one negative flanker emotion (lower emotion recognition accuracy and longer reaction times). Even though the effect was small, the implications are consistent with the previously reported ‘distractor hypothesis.’ Even more, they extend an attention capture advantage of negative emotions from targets to facial flankers. To confirm our hypothesis regarding the countervailing effect of including several negative emotions, future studies should systematically vary the number of distinct emotions within the negative emotion category.

We further tested whether congruent flanker-target combinations increased target face recognition, intensity ratings, or decreased reaction time. Analyses revealed no differences between congruent and incongruent combinations in the first two experiments, potentially due to the unbalanced composition of emotion categories. Experiment 3, however, revealed better recognition of congruent target-flanker combinations and thereby supported the ‘congruency hypothesis.’ At least in some studies, congruency effects were stronger for positive target-flanker combinations while authors found only little congruency effects for negative targets (Fenske and Eastwood, 2003; Righart and de Gelder, 2008a,b). Following this idea, we compared congruent and incongruent target-flanker combinations in each emotion of Experiment 3 (happy, fearful). While happy faces were recognized better and faster in congruent happy flankers, there was no differential influence of congruent and incongruent flankers on fearful targets, therewith, supporting earlier results. In general, congruent target-flanker combinations might predict better performance due to less response interference and a co-activation of similar neuron populations (Eriksen and Eriksen, 1974; Joubert et al., 2007; Righart and de Gelder, 2008b). Apparently, that holds for many non-affective stimuli as well as for emotions which do not require an immediate reaction (e.g., happy). Evolutionary relevant emotions though might present an exception to this. There is some evidence that threat-related information might be processed differently via a subcortical pathway bypassing primary cortical areas, potentially in order to be rapidly aware of any danger (Garrido et al., 2012; Garvert et al., 2014). Hence, in a dangerous situation the processing of threat-related stimuli might be prioritized, no matter what. However, at this point assumptions remain speculative and should be followed upon in further studies.

While the distraction effect of happy flankers was present for both genders, females rated target faces in happy flankers as less intense. This extends previous results of women being more influenced by neutral flanker stimuli (Merritt et al., 2007; Stoet, 2010) to social flankers. It further supports the assumption of females’ increased sensitivity to positive facial stimuli (Donges et al., 2012), even when the stimuli are irrelevant, as in the present study. Women also responded faster – a phenomenon previously associated with child-rearing and mother-child bonding. Our results thus support a female advantage in emotion processing (Hall, 1978; Hampson et al., 2006) and reinforce the idea that potential gender differences should be considered in any investigation of facial emotion processing.

With regard to the effects of SOA, our initial expectation was that preceding flanker presentation (SOA 300 ms, Experiment 2) would result in higher accuracy and intensity ratings of target faces compared to simultaneous presentation (SOA 0 ms, Experiment 1). However, we observed that target stimuli were rated as *more* intense when there was no delay between the flanker and the target (SOA 0 ms). We can only speculate at this point, but this could be explained, at least in part, by an amplification effect due to accumulated emotional information and a stronger contrast of the percept when multiple emotional faces are presented simultaneously (Biele and Grabowska, 2006). Yet, only a stepwise manipulation of the relative onsets of flanker and target stimuli could conclusively clarify this.

One limitation of this study, as noted, was the lack of a condition without a surrounding flanker (‘target-only’). This would have allowed an assessment of the non-specific impact of flankers, both facial and non-facial, on target face processing, something which should be addressed in future studies. Additionally, it would be interesting to assess the differential impact of all-male vs. all-female flankers on target face processing, including the potential interaction between flanker gender and receiver gender in future studies.

Summarizing our findings, in two behavioral experiments we demonstrated a reallocation of attention to unattended but salient social contexts, which influenced both emotion recognition accuracy and intensity ratings. This implies that social information is preferentially processed, even when it is located outside the attentional focus and causes distraction. We further argue that apart from gender, the emotion category composition should be considered as an important factor in studies investigating emotional context, especially when drawing conclusions about one specific emotion category. Lastly, the results of a third experiment suggest that threat-related flanker stimuli might be subject to priority-processing of evolutionary salient stimuli, which seems to ‘overrule’ a congruency effect. This might present an adaptive mechanism in order to be most sensitive to potential danger.

## Author Contributions

BSH, CR, UH, FS, and BT designed the research, BSH performed the research, BSH and CR analyzed the data, BSH, CR, and UH wrote the paper, and all authors revised the manuscript and gave final approval of the version to be published.

## Conflict of Interest Statement

The authors declare that the research was conducted in the absence of any commercial or financial relationships that could be construed as a potential conflict of interest.
