# Corrigendum: The Influence of Task-Irrelevant Flankers Depends on the Composition of Emotion Categories

**DOI:** 10.3389/fpsyg.2016.01032

**Published:** 2016-07-20

**Authors:** Barbara Schulte Holthausen, Christina Regenbogen, Bruce I. Turetsky, Frank Schneider, Ute Habel

**Affiliations:** ^1^Department of Psychiatry, Psychotherapy and Psychosomatics, Medical Faculty, RWTH Aachen UniversityAachen, Germany; ^2^JARA-BRAIN Institute 1: Structure Function RelationshipJülich, Germany; ^3^Department of Clinical Neuroscience, Karolinska InstitutetStockholm, Sweden; ^4^Department of Psychiatry, University of PennsylvaniaPhiladelphia, PA, USA

**Keywords:** threat superiority, distractor, congruency, gender differences, emotional face processing, flanker, stimulus onset asynchrony

Due to an oversight, in the original article, there was a mistake in the Author Contributions statement and CR and UH were accidentally credited with writing the paper. The corrected Author Contributions statement should read:

BSH, CR, UH, FS, and BT designed the research, BSH performed the research, BSH and CR analyzed the data, BSH interpreted the data and wrote the paper. All authors corrected the manuscript and gave final approval of the version to be published.

Furthermore, there was an error in affiliation 2 which was mistakenly published as JARA-BRAIN Institute 1: Structure Function Relationship, RWTH Aachen University, Aachen, Germany

The correct version is

JARA-BRAIN Institute 1: Structure Function Relationship, Jülich, Germany. The authors apologize for the oversights. These errors do not change the scientific conclusions of the article in any way.

## Conflict of interest statement

The authors declare that the research was conducted in the absence of any commercial or financial relationships that could be construed as a potential conflict of interest.

### Funding

This work was supported by the International Research Training Group (IRTG 1328) of the German Research Foundation (DFG). CR was supported by a post-doctoral fellowship of the German Academic Exchange Service (DAAD).

