# Supplementary material for: The Influence of Task-Irrelevant Flankers Depends on the Composition of Emotion Categories
Source: Front Psychol. 2016 May 25;7:712. doi: 10.3389/fpsyg.2016.00712 (PMC4879865; doi:10.3389/fpsyg.2016.00712)
Supplement: Supplementary file 1 [file Presentation_1.PDF]

Supplementary Material

The differential influence of task-irrelevant emotional crowds on target face processing in men and women. Schulte Holthausen B, Regenbogen C, Turetsky BI, Schneider F, Habel U.

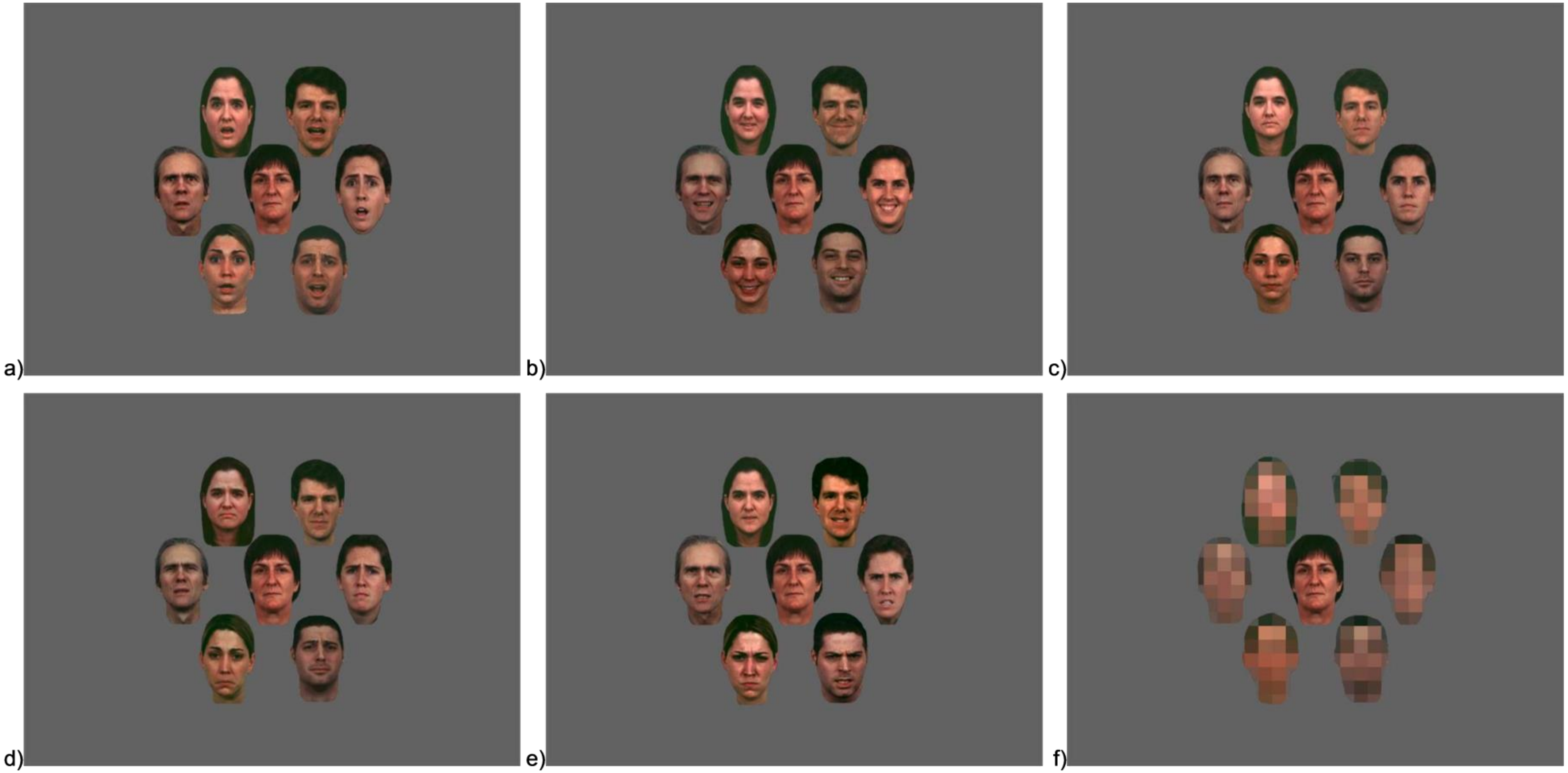

Figure S1: A neutral female target face surrounded by its corresponding a) fearful, b) happy, c) neutral, d) sad, e) angry and f) scrambled crowd.

Table S1: Pairwise comparisons of the significant main effect “crowd emotion”, of GEE model 1 analyzing emotion recognition accuracy

a. The difference is significant at the .05 level (Bonferroni-corrected)

Crowd Emotion	Crowd Emotion	Mean Difference (I-J)	95% Wald Confidence Interval (±)
Fearful	Happy	.02	0.02
	Neutral	.00	0.01
	Sad	-.01	0.02
	Angry	.00	0.02
	Scrambled	-.06[a]	0.03
Happy	Fearful	-.02	0.02
	Neutral	-.02[a]	0.02
	Sad	-.03[a]	0.02
	Angry	-.02[a]	0.02
	Scrambled	-.08[a]	0.04
Neutral	Fearful	.00	0.01
	Happy	.02[a]	0.01
	Sad	-.01	0.01
	Angry	.00	0.01
	Scrambled	-.06[a]	0.03
Sad	Fearful	.01	0.02
	Happy	.03[a]	0.01
	Neutral	.01	0.01
	Angry	.01	0.02
	Scrambled	-.05[a]	0.03
Angry	Fearful	.00	0.02
	Happy	.02[a]	0.03
	Neutral	.00	0.01
	Sad	-.01	0.02
	Scrambled	-.06[a]	0.04
Scrambled	Fearful	.06[a]	0.04
	Happy	.08[a]	0.03
	Neutral	.06[a]	0.03
	Sad	.05[a]	0.03
	Angry	.06[a]	0.03

Table S2: Pairwise comparisons of the significant main effect “crowd emotion”, of GEE model 2 analyzing average intensity ratings of correct trials.

a. The difference is significant at the .05 level (Bonferroni-corrected)

Crowd Emotion	Crowd Emotion	Mean Difference (I-J)	95% Wald Confidence Interval (±)
Fearful	Happy	.0306	0.0556
	Neutral	.0264	0.0526
	Sad	.0104	0.0594
	Angry	.0096	0.0500
	Scrambled	-.4133[a]	0.1295
Happy	Fearful	-.0306	0.0556
	Neutral	-.0042	0.0553
	Sad	-.0203	0.0542
	Angry	-.0210	0.0556
	Scrambled	-.4440[a]	0.1328
Neutral	Fearful	-.0264	0.0526
	Happy	.0042	0.0553
	Sad	-.0161	0.0658
	Angry	-.0168	0.0718
	Scrambled	-.4398[a]	0.1350
Sad	Fearful	-.0104	0.0594
	Happy	.0203	0.0542
	Neutral	.0161	0.0658
	Angry	-.0008	0.0537
	Scrambled	-.4237[a]	0.1247
Angry	Fearful	-.0096	0.0500
	Happy	.0210	0.0556
	Neutral	.0168	0.0718
	Sad	.0008	0.0537
	Scrambled	-.4229[a]	0.1206
Scrambled	Fearful	.4133[a]	0.1296
	Happy	.4440[a]	0.1327
	Neutral	.4398[a]	0.1349
	Sad	.4237[a]	0.1246
	Angry	.4229[a]	0.1207

Table S3: Pairwise comparisons of the significant main effect “experiment”, of GEE model 2 analyzing average intensity ratings of correct trials.

Experiment	Experiment	Mean Difference (I-J)	95% Wald Confidence Interval (±)
Experiment 1	Experiment 2	.2589	0.2448
Experiment 2	Experiment 1	-.2589	0.2448

Table S4: Pairwise comparisons of the significant interaction “crowd emotion” x “gender”, of GEE model 2 analyzing average intensity ratings of correct trials.

Gender	Crowd Emotion	Mean	95% Wald Confidence Interval (±)
Men	Fearful	2.7659	0.1833
	Happy	2.7879	0.1734
	Neutral	2.7111	0.1962
	Sad	2.7463	0.1765
	Angry	2.7520	0.1694
	Scrambled	3.1751	0.1966
Women	Fearful	2.9659	0.1862
	Happy	2.8827	0.1683
	Neutral	2.9679	0.1733
	Sad	2.9647	0.1701
	Angry	2.9605	0.1736
	Scrambled	3.3833	0.2031

Table S5: Pairwise comparisons of the significant main effect "crowd emotion", of GEE model 3 analyzing average reaction time of correct trials.

a. The difference is significant at the .05 level (Bonferroni-corrected)

Crowd Emotion	Crowd Emotion	Mean Difference (I-J)	95% Wald Confidence Interval (±)
Fearful	Happy	10.0059	30.0423
	Neutral	22.4624	39.6241
	Sad	20.3855	41.2013
	Angry	11.5177	29.2829
	Scrambled	-33.6480	6.5574
Happy	Fearful	-10.0059	10.0304
	Neutral	12.4565	31.6566
	Sad	10.3796	33.0196
	Angry	1.5117	22.4987
	Scrambled	-43.6539	-4.0976
Neutral	Fearful	-22.4624	-5.3006
	Happy	-12.4565	6.7437
	Sad	-2.0769	18.4344
	Angry	-10.9447	7.1883
	Scrambled	-56.1103	-17.6943
Sad	Fearful	-20.3855	0.4304
	Happy	-10.3796	12.2605
	Neutral	2.0769	22.5882
	Angry	-8.8678	14.4005
	Scrambled	-54.0334[a]	-19.2695
Angry	Fearful	-11.5177	6.2476
	Happy	-1.5117	19.4752
	Neutral	10.9447	29.0778
	Sad	8.8678	32.1362
	scrambled	-45.1656	-5.5239
Scrambled	Fearful	33.6480	73.8533
	Happy	43.6539	83.2101
	Neutral	56.1103	94.5264
	Sad	54.0334[a]	88.7974
	Angry	45.1656	84.8074

Table S6: Pairwise comparisons of the significant main effect “gender” of GEE model 3 analyzing average reaction time of correct trials.

Gender	Gender	Mean Difference (I-J)	95% Wald Confidence Interval (±)
Men	Women	86.1412	81.6534
Women	Men	-86.1412	81.6534
